# Electrospun Magnetic Nanocellulose–Polyethersulfone-Conjugated *Aspergillus oryzae* Lipase for Synthesis of Ethyl Valerate

**DOI:** 10.3390/membranes11120972

**Published:** 2021-12-09

**Authors:** Nurul Hidayah Hussin, Roswanira Abdul Wahab, Nursyafiqah Elias, Adikwu Gowon Jacob, Mohamad Hamdi Zainal-Abidin, Faizuan Abdullah, Nurul Jannah Sulaiman, Mailin Misson

**Affiliations:** 1 Department of Chemistry, Faculty of Science, Universiti Teknologi Malaysia (UTM), Johor Bahru 81310, Johor, Malaysia; nurulhidayah23289@gmail.com (N.H.H.); syafiqah.nse@gmail.com (N.E.); gjacob@fudutsinma.edu.ng (A.G.J.); mohamadhamdi@utm.my (M.H.Z.-A.); faizuan@utm.my (F.A.); 2 Enzyme Technology and Green Synthesis Group, Faculty of Science, Universiti Teknologi Malaysia (UTM), Johor Bahru 81310, Johor, Malaysia; 3 Advanced Membrane Technology Research Centre (AMTEC), Universiti Teknologi Malaysia (UTM), Johor Bahru 81310, Johor, Malaysia; 4 Department of Applied Chemistry, Federal University Dutsin-Ma (FUDMA), Dutsin-Ma P.M.B 5001, Katsina State, Nigeria; 5 Department of Bioprocess & Polymer Engineering, School of Chemical & Energy Engineering, Faculty of Engineering, Universiti Teknologi Malaysia (UTM), Johor Bahru 81310, Johor, Malaysia; jannahsulaiman@gmail.com; 6 Biotechnology Research Institute, Universiti Malaysia Sabah, Jalan UMS, Kota Kinabalu 88400, Sabah, Malaysia

**Keywords:** enzyme immobilization, nanofiber membrane, ethyl valerate, polyethersulfone, electrospinning, taguchi orthogonal design

## Abstract

A novel greener MNC/PES membrane was developed through an electrospinning technique for lipase immobilization to catalyze the synthesis of ethyl valerate (EV). In this study, the covalent immobilization of *Aspergillus oryzae* lipase (AOL) onto an electrospun nanofibrous membrane consisting of magnetic nanocellulose (MNC) and polyethersulfone (PES) to produce EV was statistically optimized. Raman spectroscopy, Fourier-transform infrared spectroscopy: attenuated total reflection, field emission scanning electron microscopy, energy dispersive X-ray spectroscopy, thermal gravimetric analysis (TGA), and differential thermal gravimetric (DTG) of MNC/PES-AOL demonstrated that AOL was successfully immobilized onto the fibers. The Taguchi design-assisted immobilization of AOL onto MNC/PES fibers identified that 1.10 mg/mL protein loading, 4 mL reaction volume, 250 rpm stirring rate, and 50 °C were optimal to yield 72.09% of EV in 24 h. The thermal stability of MNC/PES-AOL was improved by ≈20% over the free AOL, with reusability for up to five consecutive esterification cycles while demonstrating an exceptional half-life of 120 h. Briefly, the electrospun MNC/PES fibers that immobilized AOL showed promising applicability in yielding relatively good EV levels. This study suggests that using MNC as fillers in a PES to improve AOL activity and durability for a longer catalytic process could be a viable option.

## 1. Introduction

The versatility of electrospinning technology permits the generation of ultra-thin fibers, which have applicabilities in enzyme immobilization. The electrospinning process involves charging and ejecting melted polymer or solution through a spinneret under a high-voltage electric field, forming filaments of fibrous materials. The method enables the creation of highly porous, nanometer-sized materials [1]. Electrospun nanofibrous materials exhibit high porosity and large specific surface area with controllable pore size. These materials have a very high flux, which makes them ideal for supporting enzymes [2,3,4]. Moreover, electrospun materials’ customizable nature enables tuning their biocompatibility, i.e., nanoparticles [5,6,7], for different applications. They include biomedical, bioreactors, biosensors, pharmaceuticals, agricultures, drug delivery systems, electrical devices, oil remover/oil extractant, and water pollution control [8,9]. Electrospun water-soluble polymer nanofibers offer a wider range of applications, especially when coupled with enzymes. The enzymes are crosslinked into the electrospun polymeric materials, thus improving their catalytic performance [1,6]. Recent studies found that incorporating magnetic nanoparticles (MNP) into enzyme supports resulted in immobilized enzymes with enhanced stability and simple recovery processes [10,11,12].

Pertinently, the covalent attachment of enzymes onto suitable supports can avert their problematic rapid denaturation and solubility of free lipases in the reaction solution. [10,13]. As a matter of fact, several types of membranes, such as nanocellulose–silica-reinforced polyethersulfone (PES), polyvinyl alcohol, polypropylene and polyvinylidene fluoride, have been shown to be suitable for immobilizing enzymes [14]. In the case of lipase (triacylglycerol ester hydrolase, E.C. 3.1.1.3), the enzyme ranks highly among technologically relevant enzymes due to their high activity, enantio-, and regioselectivity, and also broad substrate specificity. However, extreme conditions, such as high pH, temperature, and pressure, as well as specific additives can easily render free lipases inactive. The same problem bedevils *Aspergillus oryzae* lipase (AOL), which is well-known for its high activity and selectivity as well as its relatively decent stability [15]. The lipase also rates among the cheapest and most extensively utilized in various types of different reactions [16,17]. Thus, the immobilization of AOL onto compatible supports would address these concerns while also facilitating its recovery and reuse [18,19,20].

To that end, an irreversible immobilization method is preferred since it forms covalent bonds with the electrospun fibers via crosslinkers. In fact, owing of the possibility for lower total production costs, an immobilization process with simple and fewer steps may be desirable. The multipoint covalent bonds stabilize the AOL structure to the support, minimizing allosteric inhibitions that impede catalysis. The covalent bonds reportedly interfacially activate lipases, leading to higher enzymatic activity. The same effect is expected when the AOL is crosslinked to electrospun fibers. The extra support lowers the lipases’ susceptibility to the interfering effects of reaction additives [21,22].

In this study, free AOL was conjugated to magnetic nanocelullose (MNC), which contained magnetite (Fe_3_O_4_) and polyethersulfone (PES) nanofiber composite (MNC/PES) by electrospinning technology for enhanced enzyme stability and durability for esterification. PES was the support choice of this study following its high tolerance to mechanical and chemical stresses, high surface area, ease of usage, and the possibility of different geometric configurations that have made them ideal supports for lipases [14,23]. By electrospinning the organic/inorganic materials, namely the Fe_3_O_4_ and MNC with polyethersulfone (PES), the fabricated hybrid membrane’s final characteristics could be improved for a wide-ranging application. While the hydrophobicity of the PES may prove useful in promoting the activities of immobilized lipases, the presence of the glutaraldehyde functionalized Fe_3_O_4_ and MNC serves as anchoring points for the lipases’ covalent immobilization in addition to facilitating enzyme recovery through magnetic decantation [12,21]. Meanwhile, the cellulose polymer is anchored to magnetite via carboxylate groups interacting with iron ions at the surface [24]. Hence, the same interaction was expected of that between MNC and the magnetite nanoparticles dispersed within the PES polymer. Most importantly, the study aimed to fabricate the magnetic MNC/PES membrane using a facile one-step method, as opposed to the more laborious and time-consuming multi-step preparation which we previously reported in earlier studies [14,23]. This proposed work offers a simpler preparation alternative to the conventional method to prepare a multi-component membrane. However, the lack of acceptable fabrication and immobilization procedures to fabricate the MNC/PES fibers and immobilize free AOL (MNC/PES-AOL) onto the fiber composite presented a major problem.

Hence, the objective of this study is to statistically optimize the AOL immobilization process using the Taguchi design L9 orthogonal array, aiming for a more active and operationally more stable MNC/PES-AOL. The best condition to immobilize AOL onto the MNC/PES hybrid membrane to warrant the highest percentage yield of EV remains unreported. This study highlights the first ever study detailing Taguchi Design-assisted optimization to establish the best immobilization conditions to hyperactivate and stabilize the AOL onto the MNC/PES. The efficacy of electrospun MNC/PES nanofiber composite to improve AOL stability was compared against free AOL in synthesizing ethyl valerate (EV). The biocatalyst was characterized, and its reusability was evaluated. The study hypothesized that the well-prepared MNC/PES fiber nanocomposite could activate and stabilize the AOL over its free form.

## 2. Materials and Methods

### 2.1. Materials and Chemicals

All chemicals used in this work were of analytical grade and used as received. The *A. oryzae* lipase (AOL) (≥20,000 U/mg), N-methyl-2-pyrrolidone (NMP), PES pellet (3 mm nominal granule size), ethanol (>99% purity), valeric acid (>99% purity), (3-aminopropyl), tri-ethoxysilane (APTES), n-hexane, n-heptane, ammonia, iron (III) chloride hexahydrate (FeCl_3_.6H_2_O), iron (II) chloride tetrahydrate (FeCl_2_.4H_2_O), alongside sulfuric acid (H_2_SO_4_), glacial acetic acid (99%), potassium permanganate (KMnO_4_), hydrogen peroxide and phenolphthalein were bought from Sigma-Aldrich Ltd. (St. Louis, MO, USA). Sodium hydroxide (NaOH), potassium dihydrogen phosphate (KH_2_PO_4_), dipotassium hydrogen phosphate (K_2_HPO_4_), and toluene were purchased from QReC Chemicals (New Zealand). Glutaraldehyde (25%) was purchased from ACROS Organics (New Jersey, USA), while *N*,*N*-dimethylformamide (DMF), Bradford reagent, and hydrochloric acid were acquired from V chem company (Gujarat, India). All the reagents were of analytical grade.

### 2.2. Preparation of Nanocellulose

Nanocellulose (NC) was prepared according to a reported procedure by Phanthong et al. [25] with slight modifications. Acid hydrolysis was performed by combining cellulose powder with 9 M sulfuric acid (10 mL/g cellulose) and incubating at room temperature for 5 h. Then, 20-fold ultrapure water was added to stop the reaction. The suspension was rinsed and ultracentrifuged (3500 rpm, 10 min) repeatedly till a neutral pH was achieved. The suspension was frozen overnight at −30 °C and lyophilized for 24 h to yield the NC.

### 2.3. Preparation of Magnetic Nanoparticles and Magnetic Nanocellulose

Co-precipitation was used to synthesize the magnetic nanoparticles (MNPs), in which FeCl_3_.6H_2_O (2.25 g) and FeCl_2_.4H_2_O (1.083 g) were dissolved in 100 mL ultrapure water at 80 °C under N_2_ flow and vigorous stirring. Then, 25% (*v*/*v*) ammonia solution was added dropwise to the mixture, causing the color to change from orange to black. The mixture was stirred for another 60 min. The produced MNPs were cooled at room temperature and collected by magnetic decantation using an external magnet. The MNPs were thoroughly rinsed in ultrapure water before drying in a vacuum oven at 40 °C for 10 h [26]. The mixture of MNPs (0.5 wt%) and NC (0.4 wt%) was magnetically stirred (600 rpm) in 200 mL ultrapure water and ultra-homogenized (2500 rpm) to yield the magnetic nanocellulose (MNC). The MNC was precipitated by centrifugation for 5 min at 3500 rpm and dried overnight in a vacuum oven at 35 °C [27].

### 2.4. Fabrication of Electrospun Nanofibrous Membranes

A 0.5 wt% solution of polyvinylpyrrolidone (PVP K30) was transferred to a mixture of NMP (36 wt%) and DMF (36%) [28] and stirred (100 rpm) to homogeneity. Next, 4 wt% of MNC (for the amount of PES used) was added, and the mixture was ultrasonicated (2 h) to disperse the nanomaterial. PES pellets (26 wt%) were gradually added to the mixture and magnetically stirred for 24 h to completely dissolve. The solution was degassed in an ultrasonic bath for 3 h at room temperature to eliminate trapped air bubbles.

Electrospun magnetic NC-PES was prepared by transferring the dope solution into a 10 mL rocket metal one nozzle configuration syringe pump (inner diameter = 0.37 mm). The dope solution was electrospun into nanofibers and sprayed onto aluminum foil under the following conditions: drum’s rotation speed (150 rpm), rate of injection (4 mL/h), space between the nozzle tip and collector (13–15 cm), and voltage (15 kV). Once the process was completed, the electrospun nanofibrous membranes (ENMs) were submerged in ultrapure water for 3 days to remove any residual solvent and PVP from the membranes. The ENM was air-dried for 24 h at room temperature and kept in a desiccator until further use [29].

### 2.5. Immobilization of AOL on the MNC/PES Fibers

APTES solution (1.0 mL) was added dropwise into preheated dry toluene (50.0 mL) under vigorous stirring (500 rpm). The MNC/PES fibers were then dispersed in the APTES solution and refluxed (16 h, 85 °C) with continuous stirring (180 rpm). The resulting support (AP/MNC/PES) was left to stand at room temperature (4 h) and then decanted into a round bottom flask. To remove unbound APTES, the AP/MNC/PES was rinsed with dry toluene and diethyl ether thrice, filtered and dried in a desiccator for 24 h.

The AP/MNC/PES fibers were stirred in a glutaraldehyde (Glu) aqueous solution (3.0 *v*/*v*%) for 4 h at room temperature. Then, the fibers were withdrawn from the solution, rinsed with ultrapure water, and dried overnight in a desiccator. The AOL solution was freeze-dried to obtain powdered AOL before the immobilization process. Then, the covalent immobilization of AOL onto Glu/AP/MNC/PES fibers was performed. The functional Glu/AP/MNC/PES fibers were transferred to 20 mL AOL solution (8 mg/mL) and stirred for 6 h at room temperature. The resultant MNC/PES-AOL biocatalyst was rinsed in a 0.1 M phosphate-buffered solution (pH 7.0), filtered, and dried in a desiccator (16 h) before being stored in a fridge at 4 °C [30].

### 2.6. Protein Content, Immobilization Efficiency and Lipase Activity

The amount of AOL protein in the enzyme solution before and after immobilization was estimated using the Bradford method using bovine serum albumin (BSA; 96%; Sigma-Aldrich, Steinheim am Albuch, Germany) as the protein standard [31]. The immobilized AOL protein (IP) on the MNC/PES was measured based on the difference between the initial and final protein concentrations in the prepared immobilization solution (Equation (1)). In contrast, the immobilization yield (IY) was calculated using Equation (2) [30].

(1) $$\mathrm{IP}\;\left(\frac{\mathrm{mg}}{g}\right)=\frac{\left(\mathrm{CiVi}-\;\mathrm{CfVf}\right)}{M}$$

(2) $$\mathrm{IY}\;\left(\%\right)=\frac{\mathrm{CiVi}-\;\mathrm{CfVf}}{\mathrm{CiVi}}\;\times \;100$$

where IP is the amount of AOL loaded on the support (mg protein/g support); IY is the immobilization yield (%); Ci = concentration of AOL solution before immobilization (mg/mL); Cf = concentration of AOL solution after immobilization (mg/mL); Vi = volume of AOL solution before immobilization; Vf = volume of AOL solution after immobilization; M = mass of support (g) [10,30].

Residual activity (RA) of the MNC/PES-AOL was based on the MNC/PES-AOL’s activity to free AOL in the esterification production of EV (Equation (4)). The EV was the study’s model reaction based on unsatisfactory prior attempts to synthesize the ester [32].

(3) $$\mathrm{SA}\;\left(\frac{U}{g}\right)=\frac{\left(\mathrm{Vo}\;-\;\mathrm{Vt}\right)\times \mathrm{CNaOH}}{W\;\times \;T}\times 100$$

(4) $$\mathrm{RA}\;=\frac{\mathrm{SA}\;\mathrm{of}\;\mathrm{immobilized}\;\mathrm{AOL}}{\mathrm{SA}\;\mathrm{of}\;\mathrm{free}\;\mathrm{AOL}}\times 100$$

where specific activity (SA) is calculated by dividing the number of enzyme units per mL by the protein concentration in mg/mL (Equation (3)); Vo and Vt are the initial volumes (mL) of NaOH at the time (t = 0) and the volume (mL) of NaOH at each h (t = t1, t2, t3); C_NaOH_ is the molar concentration (mol/L) of NaOH; W is the weight (g) of protein in MNC/PES-AOL, and T is time (min) of the esterification reaction. The standard enzymatic reaction was carried out in a 15 mL screw-capped vial, as reported by Jacob et al. [10], comprising valeric acid and ethanol (1:1) with stirring at 50 °C for 24 h. Equation (3) estimates lipase activity, where one international unit of activity (1 I.U.) is defined as one mol of valeric acid used in the esterification per min under assay conditions [3]. Equation (5) calculates the percentage conversion of EV and each reaction mixture triplicated.

(5) $$\mathrm{Conversion}\;\left(\%\right)=\frac{\mathrm{Vo}-\;\mathrm{Vt}}{\mathrm{Vo}}\times 100$$

where Vo = volume of NaOH at initial time; Vt = volume of NaOH at each h. Each assessed factor that resulted in the highest conversion of ethyl valerate was considered as the optimum.

### 2.7. Morphological and Structural Characterization of MNC, MNC/PES Fibers and MNC/PES-AOL Biocatalysts

Samples of NC, MNC, MNC/PES fibers, and MNC/PES-AOL biocatalysts were subjected to FTIR analysis (Perkin Elmer: Frontier 100; Waltham, MA, USA). The spectrum was recorded in transmission mode from 400–4000 cm^−1^ with a resolution of 4 cm^−1^. Next, the Raman LabRAM HR Evolution (Horiba Scientific, Kyoto, Japan) equipped with a 785 nm solid-state laser captured the spectra of MNC and MNC/PES fibers and MNC/PES-AOL between 100–2000 cm^−1^ at ambient temperature (12 mW). A 100× eyepiece microprobe focused the laser, and a charge-coupled device chamber detector identified the scattered beam with a 3 cm^−1^ spectral resolution. FESEM micrographs of MNC/PES fibers and MNC/PES-AOL were recorded on a JEOL JEM-6700F operating at 5 kV and 10 μA. The samples were first mounted on a silicon wafer and sputter-coated with a thin film of gold to avoid charging under the electron beam. Elemental compositions were determined using EDX spectroscopy. Thermogravimetric and differential thermal gravimetric thermograms of MNC and MNC/PES fibers and MNC/PES-AOL were obtained on the Thermogravimetric Analyzer (Q500-2164: Perkin Elmer, Waltham, MA, USA). Each sample was inserted into a compact ceramic alumina crucible and heated from 30 °C to 1000 °C under N_2_ flow (5 °C/min).

### 2.8. Synthesis of Ethyl Valerate Catalyzed by MNC/PES-AOL

The MNC/PES-AOL-catalyzed esterification synthesis of EV was performed in a 15 mL screw-capped bottle containing a molar ratio of ethanol:valeric acid at a molar ratio of 1:1 (Sigma Aldrich, St. Louis, MO, USA, 99%) for the free AOL or MNC/PES-AOL-catalyzed reactions. All reactions were magnetically stirred in an oil bath under varying temperatures (30, 40, 50 ℃) at stirring rates (150, 200, 250 rpm) for 24 h observation. Aliquots of the reaction mixture (200 μL) were sampled at 0 h and every 1 h intervals. Each reaction was quenched by adding 3 mL of cold acetone/ethanol (1:1), and the mixture was titrated with 0.035 M NaOH solution, using phenolphthalein as the indicator. The total acid content and lipase activity were calculated, as described by a previous study [10].

### 2.9. Taguchi L9 Orthogonal Array-Assisted Optimization of AOL Immobilization

A Taguchi L9 orthogonal array was used in this study to rapidly identify the optimal parameters for the esterification synthesis of EV by MNC/PES-AOL. Design Expert 7.1.6 software (Stat-Ease, Statistical Made Easy, Minneapolis, MN, USA) was used, involving four independent factors to be examined: the amount of protein loading (0.35, 0.73, 1.10 mg/mL), volume of reaction (4, 6, 8 mL), incubation temperature (30, 40, 50 °C), and stirring rate (150, 200, 250 rpm), with the highest conversion of EV catalyzed by the resultant MNC/PES-AOL as the response.

### 2.10. Operational Stability of MNC/PES-AOL and Free AOL

The comparison of thermal stability of free AOL and MNC/PES-AOL was performed at temperatures between 30 to 70 °C. Each enzyme was added into a reaction mixture (ethanol:valeric acid, 1:1) and assayed (50 °C, 24 h, 250 rpm, and 1.10 mg/mL protein loading) [13].

The reusability of MNC/PES-AOL was tested for consecutive cycles of the esterification synthesis of EV using the Taguchi design-identified optimal condition. After each cycle, the biocatalyst was rinsed in n-heptane thrice, dried overnight in a desiccator, and then re-dispersed in a fresh aliquot of substrates and assayed. The process was repeated until the final activity of the biocatalyst was left to 50% of the initial catalytic activity. Enzyme activity was calculated in the subsequent reactions by taking the first reaction enzyme activity as 100% [13,14].

The half-life (t_1/2_) of MNC/PES-AOL was determined using a modified procedure by Badgujar et al. [33]. The biocatalyst was incubated in n-heptane (50 °C, 120 h), and lipase activity was measured every 24 h during incubation. Finally, the leaching test followed the method reported by Abd Manan and team [18] with slight modifications. The amount of leached protein was determined using the Bradford assay [31].

## 3. Results and Discussion

### 3.1. Production of NC, MNPs and MNC

NC is a cellulose fiber derived from a variety of agricultural and municipal biomass sources. In this work, pure cellulose fibers were treated with concentrated H_2_SO_4_, which cleaves the glycosidic linkages in the amorphous domains of the biomass into smaller NC units, physically visible as a white powder [34,35]. The magnetic nanoparticles (MNPs) were subsequently synthesized using a chemical co-precipitation technique. The dark coal-colored MNPs were stirred with NC to obtain the magnetic NC (MNC). The resulting MNC was a dark brown powder compared to the MNP powder, retaining the attraction to an external magnet.

### 3.2. Fabrication of ENMs-MNC/PES Support and Immobilization of AOL on the MNC/PES Fiber

**Scheme 1:**
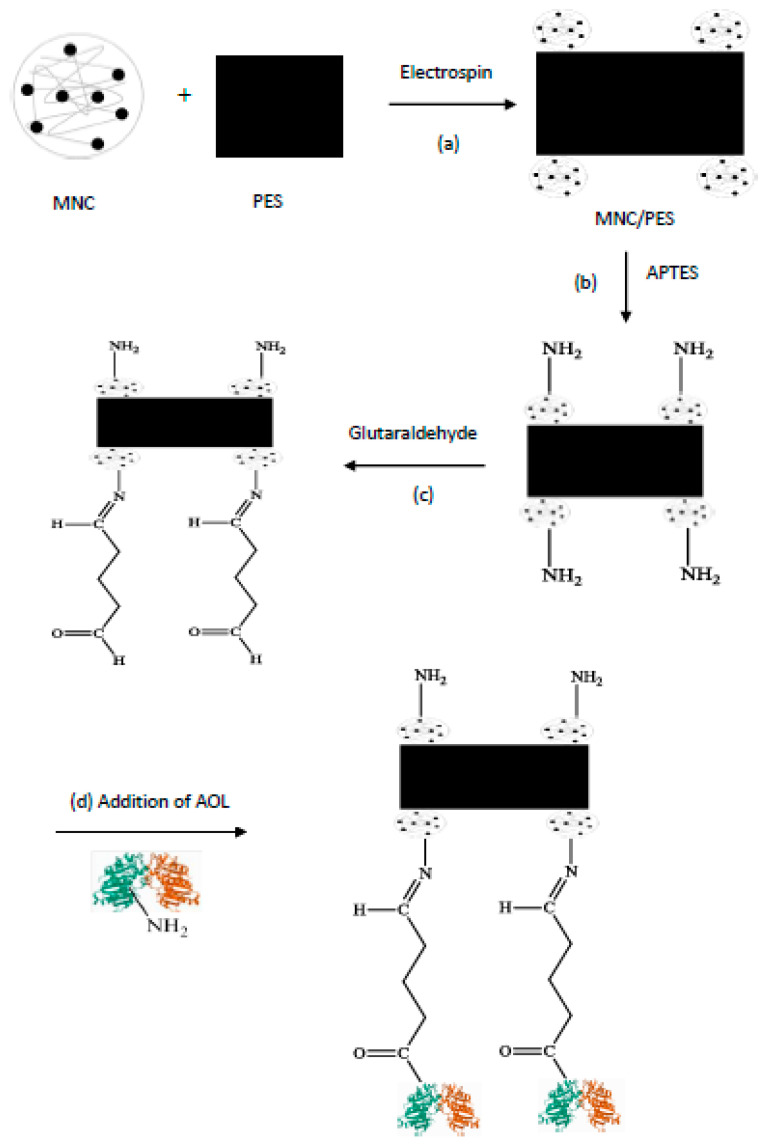
Preparation of support and the covalent attachment of AOL.

The electrospun nanofiber membranes of magnetic nanocellulose/polyethersulfone (MNC/PES) were produced in this section, integrating the MNC into a polyethersulfone (PES) solution. This technique could work to improve the characteristics of electrospun nanofibrous membranes (ENMs) to support *Aspergillus oryzae* (AOL) immobilization onto the MNC/PES. The dope solution, comprised of N-methyl-pyrrolidinone (NMP) and dimethylformamide (DMF), imparts good modularity and tensile strength to the resultant nanofibrous membrane. The study attested that enhancing fiber-within-fiber infusion during electrospinning would improve the mechanical performance of the resulting ENMs. To boost membrane anti-fouling capability and increase pore density of the MNC/PES fibers, a precise amount of hydrophilic MNC nanoparticles were introduced to the PES solution, as illustrated in Scheme 1a. Meanwhile, PES was chosen as the main polymer membrane in this study because of its large surface area and high mechanical and chemical stress tolerance. The wide pH tolerance and amenability into various geometric shapes make PES practical support for AOL immobilization. The water-soluble polymer pore former, polyvinylpyrrolidone K30 (PVP K30) in the dope solution, functions as a pore builder and bonds with PES [14].

This work used electrospun nanofibrous MNC/PES to activate and stabilize the *Aspergillus oryzae* lipase (AOL) through dative covalent bonds between the –OH groups on MNC/PES fibers and the Si atom in APTES, resulting in the AP/MNC/PES (Scheme 1b). The conjugation of AOL to MNC/PES fibers occurred through an imine (C=N) bond by the Schiff base mechanism. The nitrogen atom of carbinolamine is deprotonated with the concomitant removal of a water molecule (Scheme 1c). It is critical to avoid steric hindrance in enzyme immobilization. The study also successfully immobilized AOL onto the MNC/PES by the Schiff base mechanism, which occurred between the aldehyde groups of the modified Glu/AP/MNC/PES and the amino group AOL [10,11,36].

### 3.3. Protein Content, Immobilization Yield and Lipase Activity

In this study, approximately 88% of the protein that was initially supplied was covalently bound to the surface of electrospun MNC/PES fibers. Table 1a denotes the results of the immobilized AOL obtained in this study. As can be seen, the specific activity of MNC/PES-AOL increased substantially from 10.60 ± 0.32 U/g to 6.61 ± 0.43 U/g in the free AOL, amounting to 160% residual activity (Table 1b). The findings thus validated the positive impact of the MNC/PES membrane on AOL. The increased AOL activity might be attributed to the lipase’s primarily open conformation bonding to the MNC/PES fibers surface (active form), triggered from the interfacial activation of the AOL upon immobilization onto the fibers [4,37,38]. The hydrophobicity of PES on the MNC/PES fiber could induce the opening of AOL’s hydrophobic lid. Furthermore, the study’s findings corroborated previous studies that found covalently supported lipases to be more active than unbound lipases [4,10,38].

### 3.4. Characterization of MNC/PES and MNC/PES-AOL Biocatalyst

#### 3.4.1. FTIR:ATR Spectroscopy

**Figure 1:**
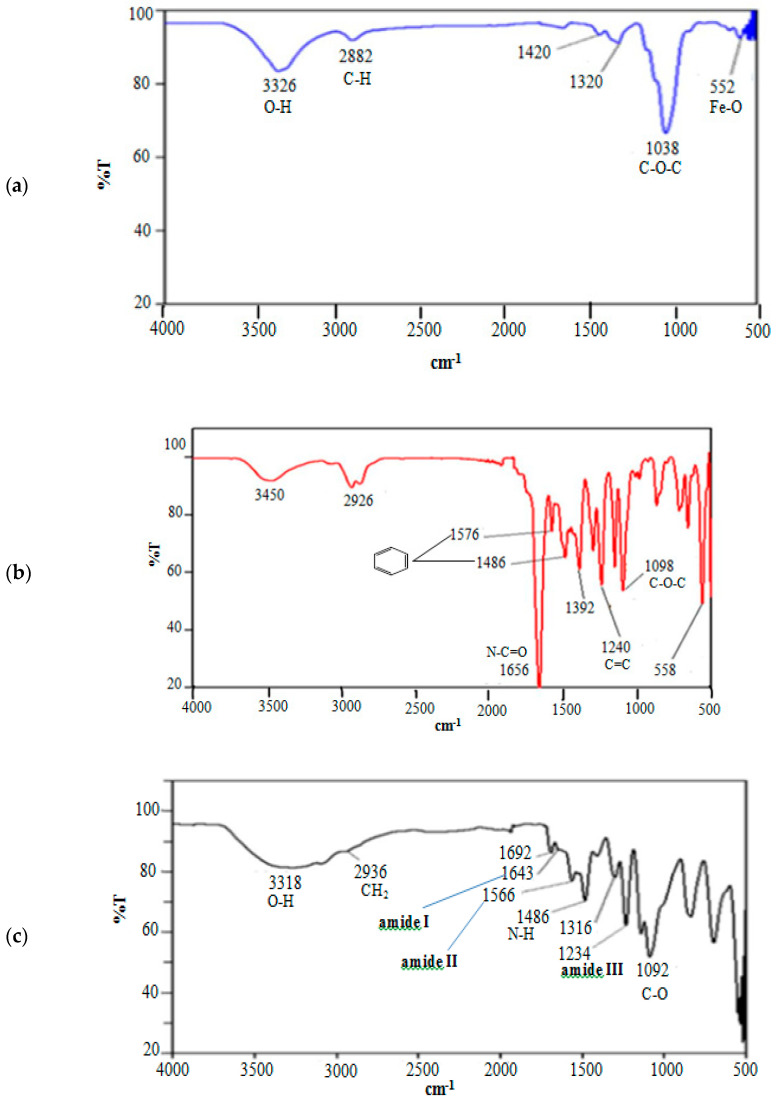
FTIR spectra of (**a**) MNC, (**b**) MNC/PES, and (**c**) MNC/PES-AOL.

Figure 1 depicts the Fourier-transform infrared spectroscopy: attenuated total reflection (FTIR:ATR) spectrum for MNC, MNC/PES fibers, and MNC/PES-AOL. The absorption peaks at 3326, 2882, 1320, 1038, and 552 cm^−1^ in the MNC spectrum were consistent with the stretching vibrations of O–H, C–H, C–O–C, and Fe–O (Figure 1a). The C–O–C stretching vibrations peak at 1038 cm^−1^ agreed with the cellulosic pyranose ring and -glycosidic bonds between glucose units. A peak for Fe–O at 552 cm^−1^ indicated that Fe_3_O_4_ (Fe^3+^ and Fe^2+^) was the dominant form [38,39,40].

The electrospun MNC/PES fiber spectrum showed typical MNC peaks (Figure 1b), with the benzene ring’s stretching vibrations seen as two peaks at 1576 cm^−1^ and 1486 cm^−1^. A peak at 1240 cm^−1^ corresponded to the stretching vibrations of C=C and aromatic ether bonds [38,41], while a prominent peak at 1656 cm^−1^ denotes the carbonyl group (N–C=O) vibration from residual NMP and PVP. A notable peak at 1098 cm^−1^ conveyed the existence of the PES C–O–C backbone [29,41].

Figure 1c for the MNC/PES-AOL spectrum revealed typical peaks of MNPs, PES, and NC, plus a minor peak of APTES at 1486 cm^−1^, from the stretching vibrations of primary N–H. Likewise, the –CH_2_ characteristic peak for the propyl group in APTES and glutaraldehyde emerged at 2936 cm^−1^. The abovementioned characteristic peaks seen in this study corresponded with previous reports on immobilized lipases [10,42]. The distinctive peaks at 1643, 1566, 1316, and 1234 cm^−1^ corresponded to amides I, II, and III of the AOL, respectively, implying the successful conjugation of AOL on the MNC/PES surface. The amide I and amide II peaks were allotted to the C=O and C=N stretching vibrations and the N–H bending mode. On the other hand, the amide III peak is the stretching vibrations of C–N and C–C and bending vibrations of N–H [10,42] (Figure 1c). A unique peak at 1092 cm^−1^ for the vibration of C–O and probably PO_4_^3−^ was observed. The latter originated from traces of phosphate buffer [43,44], used during AOL immobilization on the MNC/PES. The formation of new intermolecular hydrogen bonds between O–H molecules on AOL and functional groups on MNC/PES fibers shifted the O–H stretching vibration from 3450 cm^−1^ to 3318 cm^−1^ and increased band intensity [34,44].

#### 3.4.2. Raman Spectroscopy

**Figure 2:**
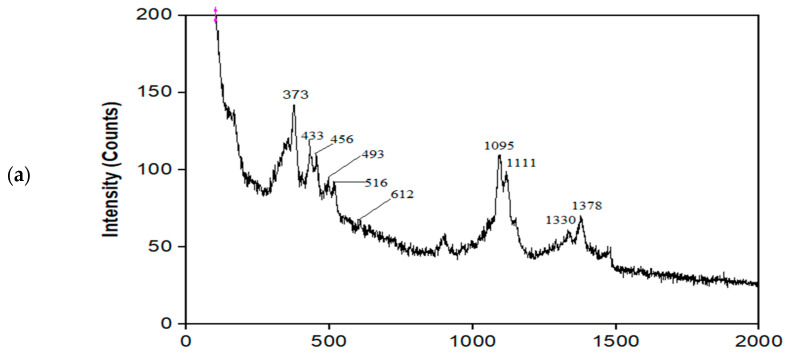

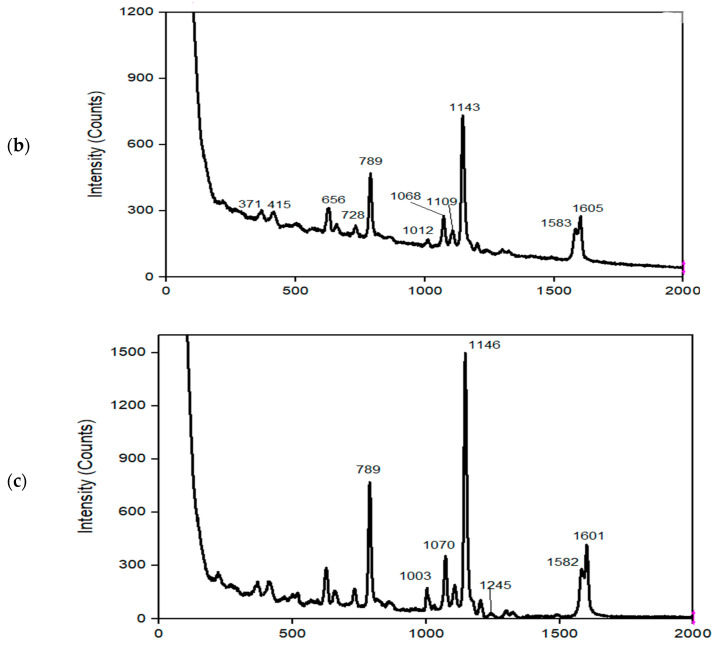
Raman spectra of (**a**) MNC, (**b**) MNC/PES, (**c**) MNC/PES-AOL.

The chemical compositions of MNC, MNC/PES, and MNC/PES-AOL were further investigated by Raman spectroscopy, and the results are presented in Figure 2. The Raman spectra of MNC (Figure 2a) showed NC and MNP bands, but with minor vibrational frequency changes. MNP bands were seen at 456, 493, 612, and 1330 cm^−1^, while bands at 456 and 493 cm^−1^ corresponded to hematite (α–Fe_2_O_3_) and maghemite (γ–Fe_2_O_3_), respectively [45]. The oxidation of MNP caused by the laser bombardment resulted in the formation of hematite and maghemite [12]. Magnetite changes to maghemite and finally to hematite when heated in the air due to an energy transfer that warms the crystalline structure [46]. However, the MNP band, which emerged at a lower wavenumber (612 cm^−1^), might be attributed to the laser treatment during analysis (a 12 mW 785 nm solid-state laser). This was consistent with the reported downshifting of magnetite vibrational mode to low-energy areas as laser intensity increases [47]. Furthermore, carbon occurs in extremely disordered graphitic forms, which explains the peak centered at 1330 cm^−1^, seen in this work [48].

The Raman vibrational bands for NC appeared at 373, 433, 516, 1095, 1111, and 1378 cm^−1^ (Figure 2a). Deformational modes of the C–C–C ring, symmetric and asymmetric stretching modes of C–O–C of the glycosidic bond emerged at 373, 1111, and 1095 cm^−1^. The C-N stretching combined with the symmetric CH_3_ bending for the amide produced a peak at 1378 cm^−1^ [34,49]. Peaks 1583 and 1605 cm^−1^ of PES (Figure 2b) presented benzene units’ conjugated C=C stretching vibration, while peaks 1068, 1070, and 1143 cm^−1^ were symmetric and asymmetric stretching modes of C–O–C. The peak for the asymmetric C–S–C stretching that appeared at 789 cm^−1^ confirmed the structure of PES in MNC/PES (Figure 2b) [34].

Peaks in the MNC/PES-AOL spectrum (Figure 2c) revealed amide I (1583 cm^−1^) and amide II bonds (1605 cm^−1^), and a CH_3_ peak of an amide and amide III for the C–N stretching band occurring at 1146 cm^−1^ and 1245 cm^−1^, respectively [10,34]. A peak at 1070 cm^−1^ complemented APTES and glutaraldehyde’s C–C aliphatic chain [12]. As a result, these data revealed that AOL existed in the MNC/PES fibers.

#### 3.4.3. FESEM-EDX

**Figure 3:**
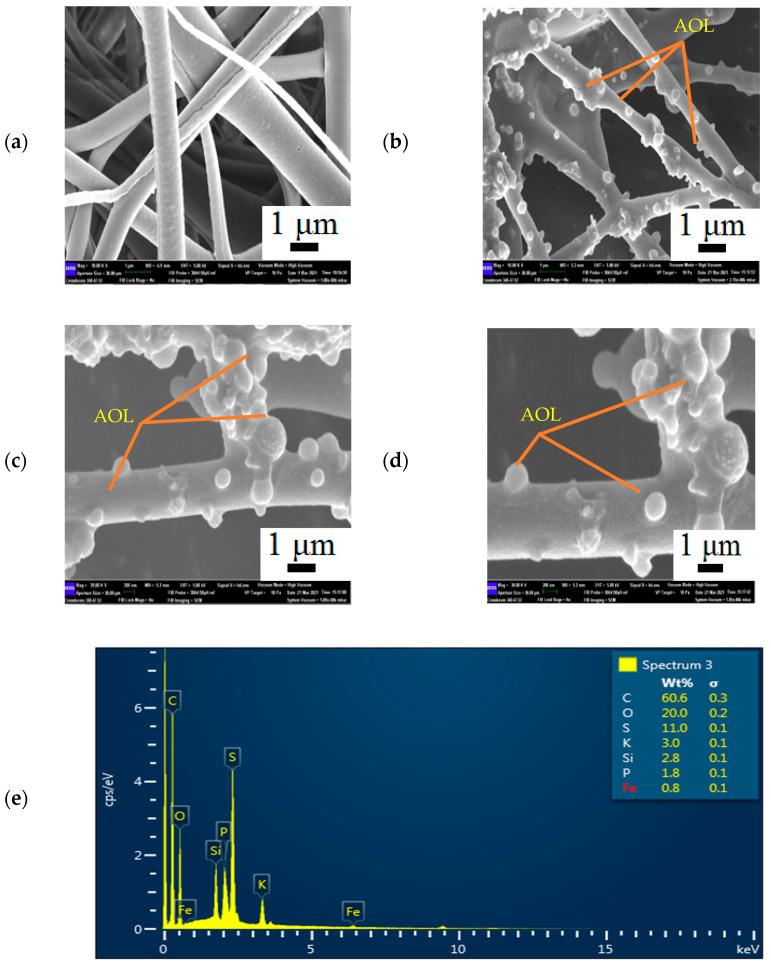
FESEM images showing the (**a**) MNC/PES at 10,000 magnification, (**b**–**d**) MNC/PES-AOL fibres before esterification at 10,000, 20,000 and 30,000 magnification, respectively, (**e**) EDX spectrum from specific region of MNC/PES-AOL fibers (reaction volume: 4 mL, incubation temperature: 50 °C, stirring rate: 250 rpm, protein loading: 1.10 mg/mL).

The field emission scanning electron microscopy (FESEM) micrographs for the electrospun MNC/PES fibers and MNC/PES-AOL are presented in Figure 3. The MNC/PES fibers (Figure 3a) revealed a relatively smooth and uniform surface, consistent with earlier studies on PES ENMs integrated with hydrous manganese dioxide and dually immobilized amylase and horseradish peroxidase (HRP) [29,50]. The surface morphology of the MNC/PES-AOL showed profound differences, reflected in a distinctively denser surface dotted with irregularly shaped white globules of AOL (Figure 3b,d). The addition of APTES and glutaraldehyde to the electrospun MNC/PES fibers produced a noticeably smoother surface as the crosslinkers interacted and silanized with the support. Similar findings were also observed for lipases immobilized on various supports [34,50].

Figure 3e illustrates the results for the energy dispersive X-ray (EDX) analysis for MNC/PES-AOL, showing 60.6 wt% carbon (C), 20.0 wt% oxygen (O), 11.0 wt% sulfur (S), 3.0 wt% potassium (K), 2.8 wt% silicon (Si), 1.8 wt% phosphorus (P), and 0.8 wt% iron (Fe). The high C content in NC and PES agreed with the high percentage weight of C in MNC/PES-AOL. In contrast, C and O originated from MNPs, NC, PES, AOL, APTES, and glutaraldehyde, while PES and APTES contributed to the S and Si peaks, respectively. The Fe, as well as P and K peaks, correspond to MNPs and the phosphate buffer used during AOL immobilization.

#### 3.4.4. TGA and DTG

**Figure 4:**
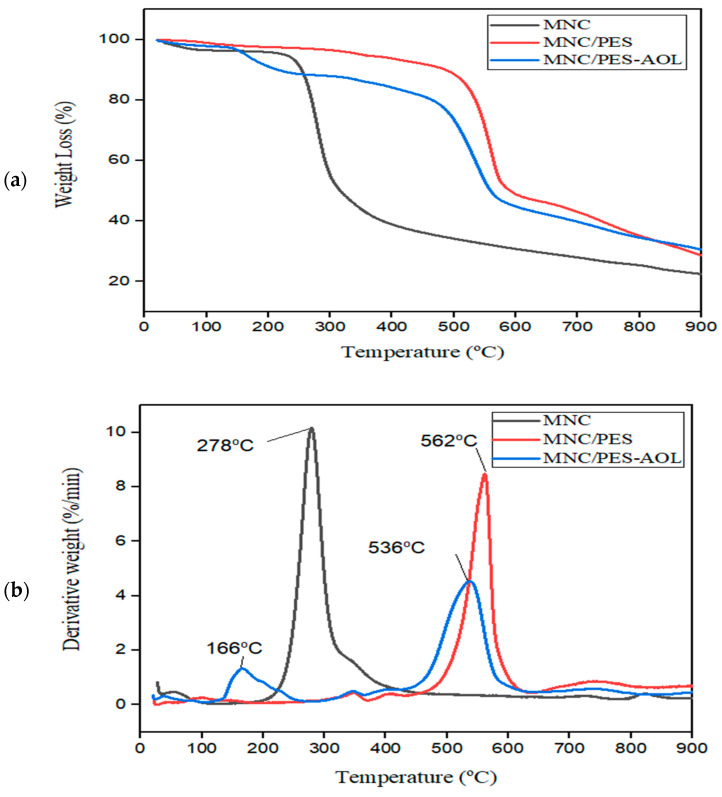
(**a**) TGA and (**b**) DTG curves for MNC, MNC/PES, and MNC/PES-AOL.

The thermal stability behavior of MNC, MNC/PES, and MNC/PES-AOL was examined by thermal gravimetric analysis (TGA), and the results are shown in Figure 4. All thermograms showed minor mass losses between 50–150 °C (Figure 4a) due to surface-bound water evaporation from the MNC/PES and the removal of residual dimethyl fluoride, seen in exothermic peaks from 40–60 °C in the differential thermal gravimetric (DTG) thermogram (Figure 4b). MNC’s thermogram indicated predominantly one thermal transition phase, with a very sharp mass drop (54.82%) between 230–400 °C due to hemicellulose and cellulose decomposition. The outcome was inferred from DTG thermograms in other studies, which showed an exothermic peak at 278 °C associated with decompositions of activated ternary alginate/NC/montmorillonite and MNC aerogels [27,51].

Decomposition of the PES backbone in MNC/PES (breaking of ether C–O bonds) appeared as a substantial mass loss (46.48%) between 500–600 °C, which corresponded to an exothermic peak at 562 °C (Figure 4b) [29]. The higher thermal degradation profile indicates thermally improved MNC/PES fibers compared to pure MNC. Conversely, the MNC/PES-AOL degradation profile exhibited two phases of deterioration patterns, between 150–250 °C (7.74%), which accompanied an exothermic peak at 166 °C, resulting from the decomposition of covalently bound AOL on the MNC/PES fibers. The MNC/PES-AOL showed a second mass loss (40.05%) between 370–600 °C, associated with an exothermic peak at 536 °C, attributable to the breakdown of NMP APTES, and glutaraldehyde [18,34]. The conjugation of AOL to the electrospun MNC/PES fibers was supported by the lower temperature of MNC/PES-AOL’s exothermic peak compared to MNC/PES.

### 3.5. Optimization of Immobilization Protocol Using the Taguchi Design

#### 3.5.1. Statistical Analysis

The L9 orthogonal array (OA) Taguchi design was used in this work to determine the optimum parameters for the esterification synthesis of EV by the MNC/PES-AOL biocatalyst. Four key reaction variables were chosen in this optimization study: reaction volume, incubation temperature, stirring rate, and protein loading during 24 h incubation to give the highest EV yield. Table 2a shows the independent variables, levels, experimental design, and actual and expected responses for the Taguchi OA, while the Design-Expert program and the model-fitting approach provided the predicated values.

ANOVA data in the optimization experiment in Table 2b demonstrated the statistical significance of the generated model, based on a low *p*-value (*p* < 0.05) and a high F-value. The estimated model’s low *p*-value (0.0003) indicated the model’s significance. Likewise, reaction volume, incubation time, and protein loading were significant in influencing the high percentage conversion of EV (*p*-value = 0.0001–0.0025), with protein loading having the greatest influence (*p*-value < 0.0001). Only the stirring rate (*p*-value > 0.05) was insignificant to the immobilization process.

Table 2a shows the comparable values of expected and actual percentage of EV conversions, which conveyed the model’s reliability to predict the best AOL immobilization condition that favors a high EV yield. This fact was echoed by the high coefficient of determination (R^2^ = 0.9903) and the comparable values of adjusted R^2^ (0.9805). The high value of adequate precision (signal-to-noise ratio) (27.767) validated the model’s adequacy to navigate the design space (>4 is desirable) (Table 2c).

#### 3.5.2. Regression of Model Equation and Analysis of Experimental Data

The regression model equation was generated using ANOVA and the contributing factors in the current investigation (Equation (6));

(6) Conversion (%)=+38.67−6.97∗A+5.31∗B+0.69∗C+18.94∗D 

where *A* represents the reaction volume, *B* represents the incubation temperature, *C* represents the stirring rate, and *D* represents the protein loading. As seen in the equation above, terms *B*, *C*, and *D* positively influence the reaction, but term *A* has the opposite effect on the AOL immobilization procedure.

**Figure 5:**
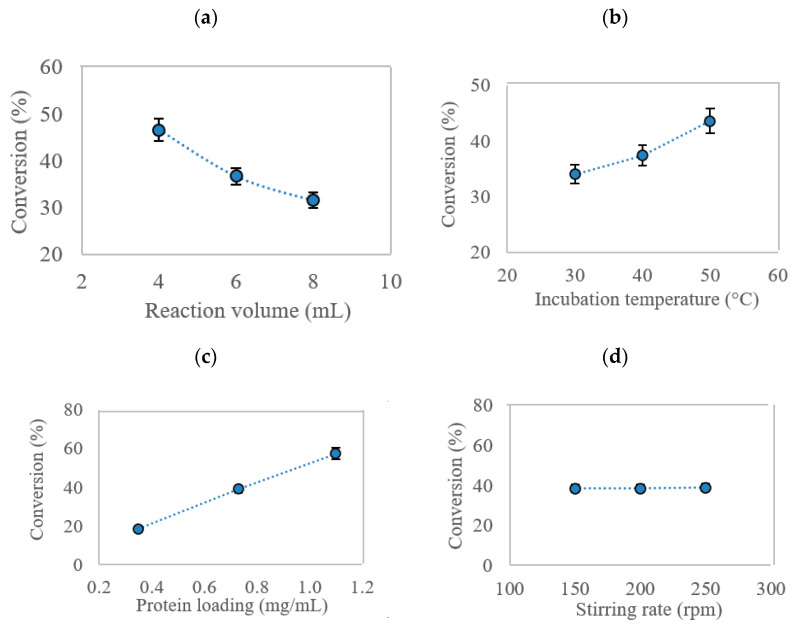
The effect of parameters (**a**) reaction volume, (**b**) incubation temperature, (**c**) stirring rate, and (**d**) protein loading on the percentage conversion of EV catalyzed by MNC/PES-AOL fibers.

Figure 5a shows the effect of reaction volume on EV conversion at three different levels (4, 6, and 8 mL) while holding other variables constant (incubation temperature: 40 °C, stirring rate: 200 rpm, protein loading: 0.73 mg/mL). At 4 mL, the MNC/PES-AOL catalyzed the highest conversion of EV at 46.6%. Due to the limited surface area of the AOL particles, the reaction yield decreased as the reaction volume grew. This resulted in less effective collisions between the MNC/PES fiber and AOL particles, likely producing MNC/PES-AOL with lower IP yield. This, in turn, reduces the reaction rate and produced ester.

The incubation temperature during immobilization is another widely accepted factor in every enzymatic system. A greater temperature can assist in increasing the system’s kinetic energy to increasing the chances of higher effective collisions [22,51]. While the MNC/PES-AOL catalyzed the highest percentage conversion of EV (43.6%) using a 6 mL reaction volume (Figure 5b), the higher reaction temperature was a plausible synergistic factor that led to the improved EV yield. The higher temperature elevated the system’s kinetic energy and increased the effective collisions between MNC/PES and AOL, to favor a higher IP of the immobilized lipase. Additionally, higher incubation temperatures reduce mixture viscosity, which aids the mixing between the support and AOL [21,52].

The effect of protein (enzyme) load is another crucial factor in producing highly effective immobilized enzymes. Figure 5c depicts the effect of protein loading in the immobilization solution to yield highly functioning MNC/PES-AOL. Increasing the protein loading had a positive effect on the EV conversion. At 1.10 mg/mL, the largest EV conversion (57.4%) was achieved, indicating an optimal AOL load for immobilization onto the electrospun MNC/PES fibers. A too-high protein load is counterproductive, leading to unwanted lipase stacking on the MNC/PES support, while a diluted lipase solution could yield MNC/PES-AOL with low lipase load. However, the study found that varying the stirring rate of the immobilization solution imparted a negligible effect on the percentage conversion of EV (Figure 5d), as seen from the large *p*-value (0.5444).

**Figure 6:**
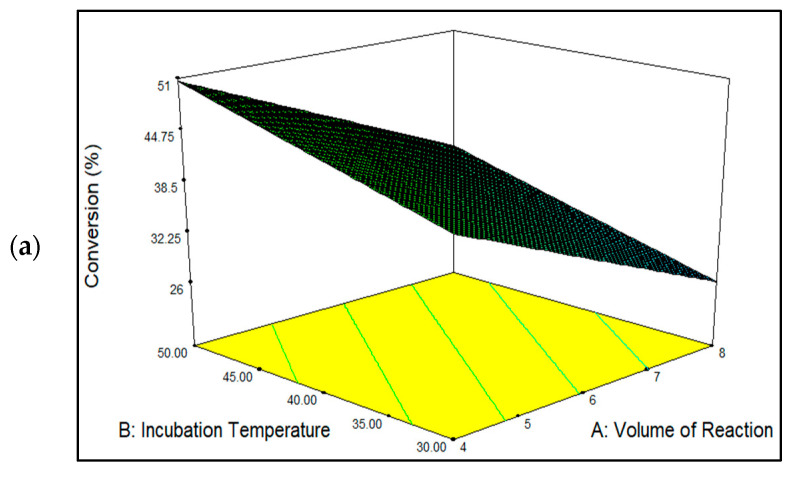

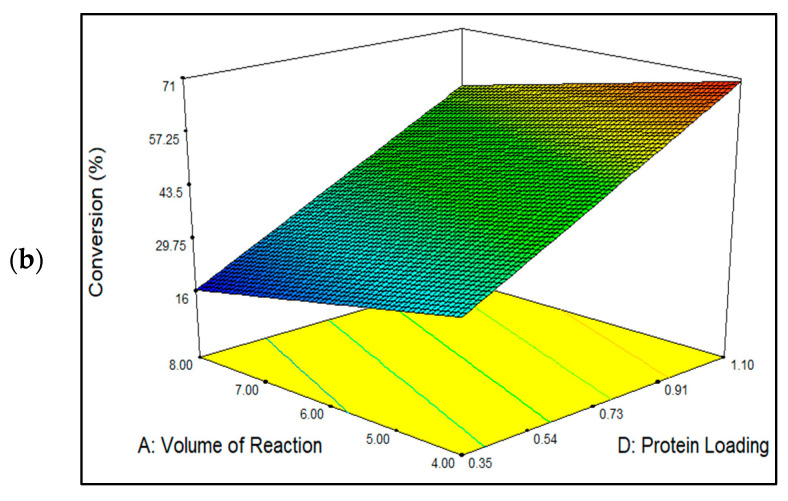
The response surface plot showing the mutual interaction for the effects of (**a**) incubation temperature and reaction volume, and (**b**) reaction volume and protein loading to influence the percentage conversion of EV catalyzed by MNC/PES-AOL fibers.

Figure 6a shows the three-dimensional plots for mutual interactions for reaction volume (*p* < 0.05) versus incubation temperature (*p* < 0.05), and Figure 6b represents the reaction volume versus protein loading (*p* < 0.0001). As seen here, raising the incubation temperature increased the percentage conversion of EV and vice versa. The best and lowest EV yields were obtained at temperatures 50 °C and 30 °C, respectively. The highest quantity of EV generated at 50 °C conveyed an optimal immobilization temperature at which the MNC/PES-AOL particles unfolded into their active state before their conjugation onto the MNC/PES fibers. Furthermore, the literature has shown that higher immobilization temperatures promote a uniform enzyme dispersion across the support before immobilization [14,53]. Hence, the same effect was predicted for free AOL before conjugation with the MNC/PES fibers. The higher temperature leads to a higher immobilization efficiency through synergistic endothermic and entropic-directed processes that alter the structure of the enzyme [14,51,53]. In contrast, the lowest EV yield occurring at a higher reaction volume was due to a larger space in the immobilization solution, which led to fewer effective collisions between the MNC/PES and AOL molecules [18] because of a more diluted mixture of AOL and MNC/PES fibers.

The mutual interaction between the reaction volumes versus protein loading is depicted in Figure 6b. It is worth noting that maximal ester conversion was achieved using the highest protein load and the smallest reaction volume. According to the results, both factors had a significant impact on developing a highly effective MNC/PES-AOL to catalyze the esterification synthesis of EV. The improved percentage of EV conversion with higher AOL loading might be explained by the greater availability of NH_2_ groups in AOL particles, which effectively collided with the C=O groups on the MNC/PES fibers to form imine bonds to bind the lipases to the support. Multipoint imine bonds increase the stiffness of immobilized AOL particles and better resist various denaturing conditions (high temperature, pressure, or additives). Instead of physical forces, the formed covalent bonds between the AOL and MNC/PES fibers, instead of physical forces, thus prevent lipase leakage during reusability and leaching tests [2,4,10]. Our findings confirmed a report by Jacob et al. [10] on the higher availability of free enzymatic active sites on the immobilized lipase, following the optimal use of enzyme loading during immobilization, concerning improvements in EV conversions.

#### 3.5.3. Optimization and Model Verification

Two verification tests were carried out to test the correctness and reliability of the Taguchi L9 OA design proposed by Design Expert 7.1.6. Table 3 presents the process parameters, including the predicted and experimental percent conversions of EV catalyzed by MNC/PES-AOL. The study attained a maximum EV conversion (72.09%) using a 4 mL reaction volume at 50 °C and a protein loading of 1.10 mg/mL (1.52% deviation). Two other validation experiments also exhibited a high degree of agreement between the predicted and actual values, alongside very low percentage deviations (0.33–0.86%). The findings affirmed that the generated model successfully explained the connections between the four tested variables and responses, as well as identifying the optimal conditions for immobilizing AOL onto the MNC/PES fibers.

### 3.6. Operational Stability of MNC/PES-AOL Membrane

#### 3.6.1. Thermal Stability

**Figure 7:**
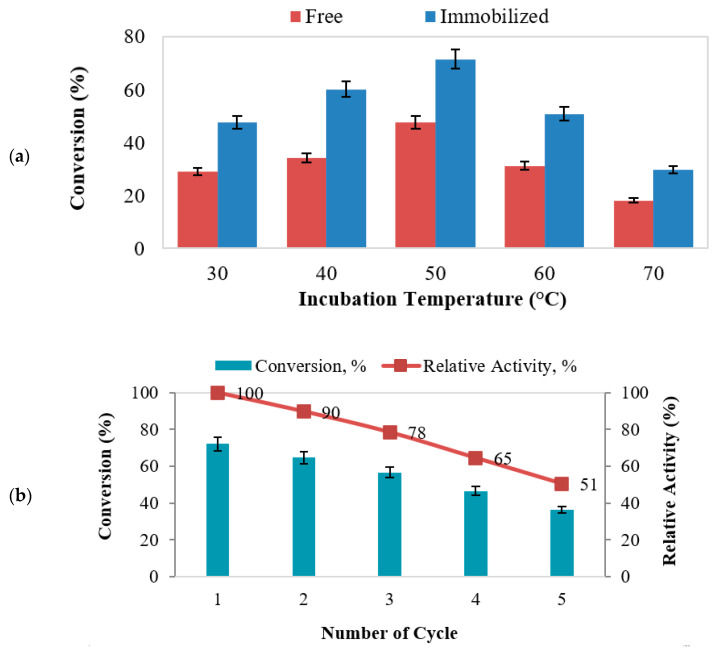

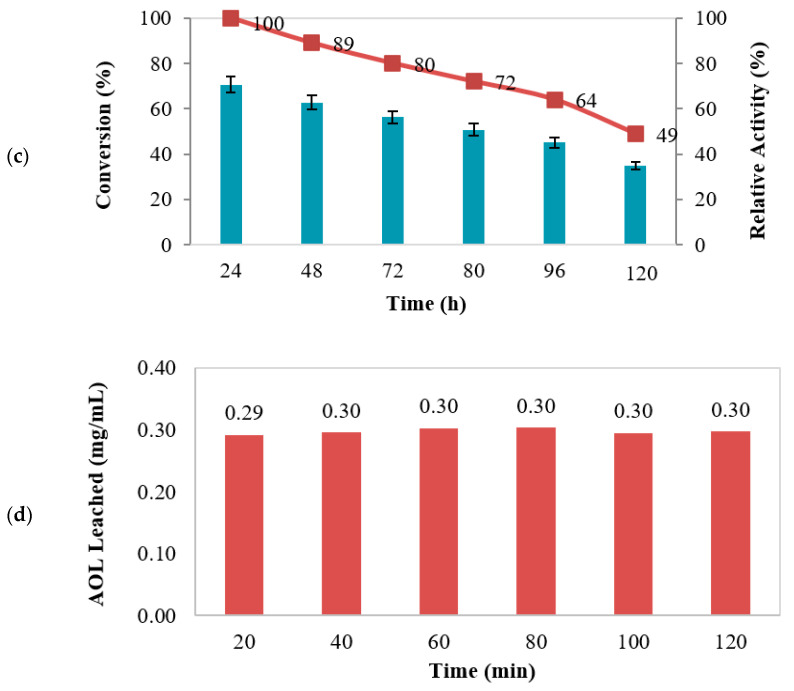
(**a**) Thermal stability (reaction volume: 4 mL, incubation temperature: 30–70 °C, stirring rate: 250 rpm, protein loading: 1.10 mg/mL), (**b**) reusability, (**c**) half-life (reaction volume: 4 mL, incubation temperature: 50 °C, stirring rate: 250 rpm, protein loading: 1.10 mg/mL) of MNC/PES-AOL on the synthesis of EV, and (**d**) AOL lipase leaching test under optimal reaction conditions.

In this study, the thermal stability of free AOL and MNC/PES-AOL biocatalysts was compared for temperatures between 30–70 °C to enable the highest percentage conversion of EV. Figure 7a depicts the measurement of thermal activity for both free and immobilized AOL. According to the study, the percentage yield of ester for the free and immobilized AOL-catalyzed reactions increases gradually as the temperature rises from 30 to 50 °C. However, increasing the temperature to 70 °C rendered the esterification process ineffective, as demonstrated by the reduced degree of ester conversion. For both free and immobilized AOL, 50 °C led to the maximum conversion of EV at 47.5% and 71.5%, respectively.

Furthermore, the electrospun MNC/PES improved the thermal stability of AOL due to the new multipoint bonds [51,54]. The added intermolecular bonds rigidified the AOL structure to prevent premature unraveling [4]. Nevertheless, further raising the temperature above 50 °C reduced the percentage of EV due to the higher temperature that could deactivate the lipase. The results established that the shielding effect of MNC/PES on AOL against thermal denaturation was effective for temperatures up to 50 °C, verifying the MNC/PES fiber’s efficacy in stabilizing AOL at higher temperatures.

#### 3.6.2. Reusability

**Figure 8:**
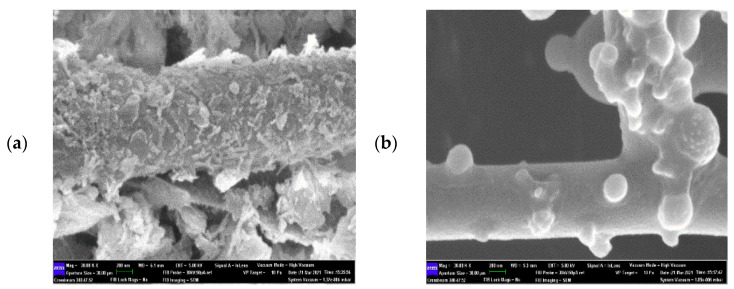
FESEM images at 30,000× magnification showing micrographs of (**a**) MNC/PES-AOL before esterification; and (**b**) MNC/PES-AOL fiber after 5 successive cycles (reaction volume: 4 mL, incubation temperature: 50 °C, stirring rate: 250 rpm, protein loading: 1.10 mg/mL).

Reusability focuses on the ability of immobilized lipase for repeated usage to retain more than 50% of initial activity [12,13]. It is one of the most important elements driving the growing use of immobilized lipases in industrial sectors and augments operational- and financial viability [55,56]. Figure 7b denotes a reduction in MNC/PES-AOL activity, with reusability up to 5 cycles. The gradual reduction in EV production was due to depositions of substrates (ethanol/valeric acid) or formed EV over the biocatalyst’s surface that interfered with catalysis [12,13,57]. A close examination of the reused MNC/PES-AOL (Figure 8a) showed a coarser surface morphology over the freshly prepared ones (Figure 8b). The former shows visible cracks or chunks, likely damage due to mechanical stress from frequent stirring and washing steps. Contributions from the accumulation of surplus water and AOL denaturation due to repeated uses were also possible, consistent with descriptions of immobilized enzymes in other studies [51,57,58]. Based on the above findings, the robustness of MNC/PES-AOL is sufficient for multiple rounds of reuse in EV production.

#### 3.6.3. Half-Life

The half-life of immobilized lipase refers to the duration that reduces enzyme activity by 50% over its initial levels [59]. In this study, the half-life of MNC/PES-AOL was examined by incubation in n-heptane at 50 °C for 120 h, and the data are illustrated in Figure 7c. There was a gradual reduction in esterification activity as the incubation period was increased from 24 to 120 h. The prepared MNC/PES-AOL maintained 49% of their initial operation after 120 h, giving them a half-life of 119 h. Due to the high half-life established by MNC/PES-AOL fiber efficacy, AOL was rigidized through its multipoint covalent attachment to the MNC/PES support.

The result of this study was consistent with the reported findings by earlier research [19,60] for immobilized *Pseudomonas cepacia* lipase (PCL) and CRL. The lipases had half-lives of 130 and 88 h, respectively. Badgujar et al. employed a biodegradable ternary combination of polylactic acid, chitosan, and polyvinyl alcohol to immobilize the PCL [60]. On the other hand, carboxymethyl cellulose and chitosan-based co-polymer cross-linked with glutaraldehyde was used as the support matrix for CRL immobilization. According to the findings, the MNC/PES fibers appeared to give stabilizing support, delaying the deactivation of AOL over long-term storage. The results also suggest that MNC/PES fibers could support immobilizing different types of lipase.

#### 3.6.4. Leaching

Leaching is the most common cause of activity loss in successive cycles, especially when enzyme loads are large, which increases the potential of enzymes to be physically adsorbed to the supports [61,62]. The stirring of MNC/PES-AOL for 20 min in phosphate buffer produced 0.29 mg/mL of leached AOL, while a further 40 min and 1 h stirring caused a marginal increase in protein content to 0.30 mg/mL. This outcome indicated that after 40 min of stirring, only covalently bonded AOL molecules remained on the MNC/PES fiber beyond 40 min of stirring (Figure 7d). The initially leached AOL protein was due to physically adsorbed AOL on the MNC/PES fibers because of insufficient rinsing in the last immobilization step [18]. Abd Manan and his team [18] also recorded 0.03 mg/mL (pH 7.0) of leached *Candida rugosa* lipase from the chitosan-chitin nanowhiskers support in the 1 h of agitation in phosphate buffer, as well as other similar studies [11,63]. Hence, the results conveyed the procedure’s suitability to covalently immobilize free AOL onto the electrospun MNC/PES fiber.

### 3.7. Comparative Study of Ethyl Valerate Production Using Other Enzymes

In this study, immobilizing AOL onto the MNC/PES resulted in the MNC/PES-AOL being capable of producing 72.09% of EV in 24 h. This value is higher than CRL immobilized onto poly(o-toluidine) functionalized magnetic nanocomposite (Fe_3_O_4_@POT) (65%) [32], but lower than the commercial lipase Novozyme 435 (69.2% EV, 40 min, 50 °C) microwave-assisted synthetic reaction. The *Thermomyces lanuginosus* polyhydroxybutyrate immobilized lipase (PB-TLL) synthesized 92% EV in 105 min at 30.5 °C, using 18% m/v of the biocatalyst [64]. Understandably, EV yields were high for the Novozyme 435- and PB-supported TLL because of the high enzyme loadings used in the synthesis. Khoobi and his colleagues [65] synthesized TLL conjugated to MCM-41@PEI-GLU, which showed good activity to produce 79.1% EV in 24 h, with n-hexane as the solvent. The *Burkholderia cepacia* lipase (BCL) immobilized on sodium alginate produced ≈90% EV in 120 h [66]. Asmat and his team [67] immobilized CRL onto a support made of magnetic graphene-anchored silica nanocomposite and achieved a 90% EV production in 24 h. It can be construed that the electrospun MNC/PES fiber improved the enzymatic activity and operational stability of immobilized AOL, thus producing higher yields of EV compared to its free counterparts and other immobilized enzymes.

## 4. Conclusions

In summary, our study effectively established the fabrication of catalytically efficient MNC/PES fibers as well as the optimal immobilization conditions for conjugating the AOL for high-yield EV synthesis. The catalytic efficiency of MNC/PES-AOL catalyzed EV synthesis was optimized using the Taguchi technique. The biocatalyst was best immobilized by incubating 1.10 mg/mL of MNC/PES-AOL in 4.0 mL reaction volume at 50 °C. In 24 h, the biocatalyst effectively produced 72.09% of EV. Remarkably, the synthesized MNC/PES-AOL was operationally stable for five consecutive esterification cycles, with exceptional thermal stability and a half-life of 120 h. Hence, it was demonstrated that the electrospun MNC/PES fibers could improve AOL activity and durability for a prolonged and repeated catalytic process to sustainably synthesize EV.

## Figures and Tables

**Table 1:** (**a**) Calculation of immobilized AOL protein using the Bradford method, and (**b**) the specific and residual activities of free AOL and MNC/PES-AOL.

**(a)**	**Immobilization Parameter**			
	Total working volume (mL)		20.0	
	Initial lipase content (mg)		0.0722	
	Final lipase content (mg)		0.008	
	Difference in protein content (mg)		0.0642	
	Mass of support (g)		1.0	
	Immobilized protein (mg/g)		7.11	
**(b)**				
**No**	**Type of Biocatalyst**	**Protein Concentration (mg/mL)**	**Specific Activity (U/g)**	**Residual Activity (%)**
1	Free AOL	7.11	6.61 ± 0.43	100
2	MNC/PES-AOL	7.11	10.60 ± 0.32	160

**Table 2:** (**a**) Experimental conditions for the various runs of the Taguchi design for the actual and predicted responses, (**b**) ANOVA of parameters affecting the immobilization process, and (**c**) the ANOVA of statistical parameters.

**(a)**						
**Run**	**Reaction Volume (mL)**	**Incubation Temperature (°C)**	**Stirring Rate (rpm)**	**Protein Loading (mg/mL)**	**Actual Conversion (%)**	**Predicted Conversion** **(%)**
1	8	30	150	1.10	49.62	49.95
2	4	50	200	0.73	45.56	45.63
3	4	40	250	1.10	72.09	70.57
4	8	40	200	0.35	19.20	18.08
5	6	50	150	0.73	42.50	43.29
6	4	40	150	0.35	22.22	20.70
7	6	50	250	0.35	17.11	20.41
8	6	30	200	1.10	50.44	52.29
9	8	30	250	0.73	29.25	27.07
**(b) Source**		**Sum of Squares**	**Degree of** **Freedom**	**Mean Square**	***F*** **-Value**	***p*** **-Value** **(Prob > F)**
Model		2615.00	4	653.75	101.63	0.0003 *
Reaction Volume		291.21	1	291.21	45.27	0.0025
Incubation Time		169.39	1	169.39	26.33	0.0068
Stirring Rate		2.82	1	2.82	0.44	0.5444
Protein Loading		2151.58	1	2151.58	334.48	<0.0001 *
Residual		25.73	4	6.43		
Corrected Total		2640.73	8			
**(c)** Standard Deviation			2.54	R^2^	0.9903	
Mean			38.67	Adjusted R^2^	0.9805	
Coefficient of Variance (%)			6.56	Predicted R^2^	0.9307	
PRESS			182.90	S/N Ratio	27.767	

* Note: Highly significant.

**Table 3:** Validation of the model for optimum conditions for MNC/PES-AOL-catalyzed synthesis of EV.

No	Parameters				EV Conversion (%)		
	A (mL)	B (°C)	C (rpm)	D (mg/mL)	Predicted	Actual	Deviation
1	4	50	250	1.10	70.57	72.09	1.52
2	4	48.05	250	1.10	69.80	68.94	0.86
3	6	30	200	1.10	52.29	51.96	0.33

## Data Availability

The raw/processed data required to reproduce these findings cannot be shared at this time as the data also form part of an ongoing study.
